# The Impact of Body Mass Index on Survival and Clinical Outcomes in Pulmonary Arterial Hypertension: Revisiting the Obesity Paradox

**DOI:** 10.7759/cureus.95467

**Published:** 2025-10-26

**Authors:** Angel Yazdi, Andrea Ramirez, Robert E Walter, Prangthip Charoenpong

**Affiliations:** 1 Pulmonology and Critical Care, Baylor College of Medicine, Houston, USA; 2 Internal Medicine, Louisiana State University (LSU) Health Shreveport, Shreveport, USA; 3 Pulmonology and Critical Care, Louisiana State University Health Sciences Center, Shreveport, USA; 4 Pulmonology and Critical Care, Louisiana State University (LSU) Health Shreveport, Shreveport, USA

**Keywords:** obesity, obesity paradox, pulmonary arterial hypertension, pulmonary hypertension, survival

## Abstract

Objective: This study aims to investigate the impact of body mass index (BMI) on survival outcomes and clinical characteristics in patients with pulmonary arterial hypertension (PAH). Given the conflicting evidence on the "obesity paradox," we sought to further explore this phenomenon within a demographically diverse cohort at Louisiana State University Health Sciences Center in Shreveport.

Materials and methods: We conducted a retrospective observational study of PAH patients aged 18 years and older who received care at our institution from 2019 to 2022. Patients were categorized by BMI as normal (<25 kg/m²), overweight (25-30 kg/m²), and obese (>30 kg/m²). Clinical and demographic data, including age, gender, comorbidities, PAH subtype, and functional measures, were collected. Hemodynamic parameters, right heart catheterization (RHC) findings, and functional assessments were also included. Survival analysis was conducted using the Kaplan-Meier method, with group comparisons performed via ANOVA and chi-squared tests.

Results: A total of 69 patients were included, with 55.1% classified as obese, 26.1% as overweight, and 18.8% as having a normal BMI. The majority of patients were female (72%) and African American (44.8%). Although obese patients displayed more severe hemodynamic measures, including higher mean pulmonary artery pressure (mPAP) and greater right ventricular dysfunction, the survival analysis showed that the obese group had a non-significant trend toward better survival outcomes compared to normal BMI and overweight groups. Functional improvements on the 6-minute walk test (6MWT) post-treatment were also more pronounced in the obese group.

Conclusion: Our findings contribute to the understanding of the obesity paradox in PAH, revealing a potential trend toward improved survival in obese PAH patients despite more adverse hemodynamic profiles. These results underscore the need for further research to elucidate the mechanisms underlying the obesity paradox in PAH and to determine if BMI-targeted management strategies may benefit this population.

## Introduction

With the growing obesity epidemic, there is an increase in the prevalence of associated diseases, particularly diabetes, cardiovascular disease, and cancer [1]. Obesity is also linked to sleep disorders and hypoventilation, which can impact pulmonary circulation and contribute to pulmonary hypertension [2].

Pulmonary hypertension (PH) is currently defined by an elevation in the mean pulmonary arterial pressure (mPAP) greater than or equal to 20 mmHg at rest [3]. PH is subdivided into five mechanistically and histopathologically associated groups. Specifically, pulmonary arterial hypertension (PAH) is hemodynamically defined as mPAP greater than 20 mmHg, pulmonary capillary wedge pressure (PCWP) ≤ 15, and pulmonary vascular resistance (PVR) ≥ 2 wood units, in the absence of other causes of pre-capillary PH. These precise parameters are measured via the right heart catheterization (RHC) [4].

Data from the Registry to Evaluate Early and Long-Term PAH Disease Management (REVEAL) study shows that 32% of PAH patients are obese [5]. The proposed mechanisms of obesity contributing to the development of PAH are complex and heterogeneous [6]. Excess adipose tissue leads to chronic low-grade inflammation and insulin resistance, which may contribute to the pathogenesis of PAH [2]. Oxidative stress due to obesity has also been linked to morphological changes in the lung vasculature and contributes to PAH development [7]. Other endocrine abnormalities in obesity, such as increased leptin, decreased adiponectin, and altered estrogen metabolism, have also been associated with PAH [8]. Functionally, obesity has been linked to worse outcomes on the 6-minute walk test (6MWT) and exercise tolerance, parameters used for PAH assessment [9].

Despite the association between obesity and PAH, there is growing evidence of the "obesity paradox," where obese patients have better long-term outcomes of PAH. This may stem from the contribution of obesity to subclinical right ventricular (RV) dysfunction, which paradoxically preserves RV function once the patient develops PAH [10]. Another hypothesis involves PAH treatment, which results in a reduction in oxidative stress and proinflammatory cytokines, which targets the culprit of PAH in obese patients [11]. Data from the REVEAL study also supports this evidence by showing a reduced risk of death and survival benefit in obese patients with PAH [5,12].

Unlike the REVEAL study, both the Scottish Pulmonary Vascular Unit (SPVU) [9] and the French Pulmonary Hypertension Network registry [13] presented data that indicated no significant association between body mass index (BMI) and mortality in PAH patients, challenging the notion of the obesity paradox.

In light of the escalating global obesity epidemic and its intricate connections to various diseases, gaining insight into its specific impact on the survival rates of PAH patients is imperative, especially considering the conflicting findings from previous studies. The interplay between obesity and PAH outcomes remains a critical area requiring deeper investigation. Our study, conducted at Louisiana State University Health Sciences Center in Shreveport, seeks to investigate this phenomenon further within a distinct cohort characterized by a high prevalence of obesity [14], a wide range of comorbidities, and diverse demographics. This unique population offers valuable insights into how BMI and associated factors may influence PAH outcomes, particularly among patient groups underrepresented in existing research. We anticipate that our findings will enhance understanding of the complex relationship between BMI and survival in PAH, ultimately contributing to improved, tailored care approaches for this challenging patient population.

## Materials and methods

This study was conducted in compliance with ethical standards set by the Institutional Review Board (IRB) at Louisiana State University Health Sciences Center in Shreveport and in accordance with the Declaration of Helsinki. IRB approval (protocol number STUDY00001858) was obtained before initiating this retrospective study. Due to the use of de-identified patient data, the IRB granted a waiver of informed consent. All collected data were anonymized to protect participant confidentiality.

Study design and patient characteristics

This is a retrospective observational study conducted at Louisiana State University Health Sciences Center in Shreveport, LA. Patients above 18 years of age with a diagnosis of PAH who received care at our institution from 2019 to 2022 were identified via electronic medical records. Patients were then divided into normal BMI (BMI <25 kg/m²), overweight (BMI 25-30 kg/m²), and obese (BMI >30 kg/m²) categories. Data were collected from the initial presentation and approximately three months of follow-up after the initiation of PAH treatment. Patients who did not follow up at our institution after initiating treatment were excluded from the study.

Measurements

Baseline characteristics included age, gender, race, weight, height, BMI, and comorbidities. Social history, such as the use of tobacco, alcohol, and recreational drugs, was also obtained.

Further determinants, such as PAH subgroups, PAH treatment including pulmonary vasodilators and diuretics, natriuretic peptide concentrations (BNP/pro-BNP), New York Heart Association (NYHA) class, 6MWT, and REVEAL lite score, were also collected. BNP was converted to pro-BNP using the conversion formula published by Kasahara et al. [15]. Hemodynamic findings were obtained from echocardiography and RHC.

Statistical analysis

Patients were categorized into three BMI groups as described above. Continuous variables were presented as mean ± standard deviation (SD) for normally distributed data or median with interquartile range (IQR) for non-normally distributed data. Categorical variables were summarized as counts and percentages. Comparisons of baseline demographics and clinical characteristics (including symptoms, functional class, 6MWT, REVEAL lite score, and hemodynamics at diagnosis) across the BMI groups (<25, 25-30, and >30 kg/m²) were conducted using ANOVA with post hoc pairwise comparisons for continuous variables and chi-squared or Fisher's exact test for categorical variables, as appropriate. All analyses were performed using STATA version 14.2 (StataCorp. 2016. Stata Statistical Software: Release 14. College Station, TX: StataCorp LLC).

Survival outcomes were analyzed using the Kaplan-Meier (K-M) method, with comparisons across the three BMI groups conducted via the log-rank test. A survival cutoff date of August 1, 2023, was applied to ensure consistency in the follow-up period across all participants.

For handling missing data, we used a complete-case analysis approach. Individuals with incomplete data for a specific variable were excluded from analyses involving that variable but remained in the dataset for analyses where their data were complete.

## Results

Patient cohort

We identified 69 PAH patients for our study, with 50 of them being female. Among the participants, 30 (44.78%) were African American, and the average age in the cohort was 56.3 years. These patients were categorized into BMI groups: 13 (18.8%) with normal BMI, 18 (26.1%) overweight, and 38 (55.1%) obese. Additionally, we observed comorbidities within the cohort, including nine (13%) cases of diabetes mellitus (DM), 36 (52.2%) cases of hypertension (HTN), nine (13%) diagnoses of coronary artery disease (CAD), and 17 patients (24.6%) with obstructive sleep apnea (OSA).

There was a female predominance across all BMI groups, with 76.3% in the obese group, 61.1% in the overweight group, and 76.9% in the normal BMI group. The most prevalent subgroup of PAH in our cohort was idiopathic pulmonary arterial hypertension (IPAH), accounting for 30 (43.48%) cases, followed by connective tissue disease-associated PAH (CTD-PAH) at 24 (34.78%) and methamphetamine-associated PAH (MA-PAH) at eight (11.59%). The highest prevalence of IPAH is observed in the obese group at 47.4%. Conversely, within the normal BMI group, the prevalence of CTD-PAH is most prominent, constituting 61.5% of our patient population. In terms of OSA incidence, the obese group exhibits the highest rate at 31.6%, surpassing the rates in the normal BMI (7.7%) and overweight (22.2%) groups, although this difference does not reach statistical significance. No statistically significant differences were observed in the prevalence of DM, HTN, CAD, and chronic kidney disease (CKD) among the three BMI groups. Similarly, there is no statistically significant distinction in the rates of smoking, alcohol use, or recreational drug usage across the three groups (Table 1).

**Table 1:** Comparison of baseline characteristics of PAH patients with normal BMI, overweight, and obesity ^†^Fisher's exact test ^‡^One-way ANOVA Chi-square test. Significant if p<0.05 BMI: body mass index; DM: diabetes mellitus; HTN: hypertension; CAD: coronary artery disease; OSA: obstructive sleep apnea; CKD: chronic kidney disease; PAH: pulmonary arterial hypertension; IPAH: idiopathic pulmonary arterial hypertension; CTD-PAH: connective tissue disease-associated PAH; MA-PAH: methamphetamine-associated PAH; HIV-PAH: human immunodeficiency virus-associated PAH; CHD-PAH: congenital heart disease-associated PAH; NYHA: New York Heart Association; FVC: forced vital capacity; FEV1: forced expiratory volume in one second; DLCO: diffusing capacity for carbon monoxide; IV: intravenous; SQ: subcutaneous

Variables	Total (n=69)	BMI <25 (n=13)	BMI 25-30 (n=18)	BMI >30 (n=38)	p-value
Age, Mean ± SD	69 (56.3 ± 13.6)	13 (54.9 ± 17.5)	18 (59.3 ± 14)	38 (55.4 ± 12)	0.56^‡^
Sex (Female: n, %)	50 (72.5%)	10 (76.9%)	11 (61.1%)	29 (76.3%)	0.47^†^
BMI, Mean ± SD	69 (31.9 ± 7.6)	13 (22.6 ± 1.9)	18 (27.6 ± 1.5)	38 (37.2 ± 5.9)	<0.001^‡^
Race (n, %)					
Caucasian	36 (53.7%)	5 (38.5%)	12 (66.7%)	19 (52.8)	-
African American	30 (44.8%)	8 (61.5%)	6 (33.3%)	16 (44.4%)	-
Other	1 (1.5%)	0	0	1 (2.8%)	-
Comorbidities (n, %)					
DM	9 (13%)	1 (7.7%)	0	8 (21.1%)	0.08^†^
HTN	36 (52.2%)	6 (46.2%)	10 (55.6%)	20 (52.6%)	0.87
CAD	9 (13%)	2 (15.4%)	1 (5.6%)	6 (15.8%)	0.62^†^
OSA	17 (24.6%)	1 (7.7%)	4 (22.2%)	12 (31.6%)	0.21^†^
CKD	10 (14.5%)	1 (7.7%)	3 (16.7%)	6 (15.8%)	0.81^†^
Smoking	27 (39.1%)	7 (53.9%)	5 (27.8%)	15 (39.5%)	0.34
Alcohol	16 (23.2%)	4 (30.8%)	5 (27.8%)	7 (18.4%)	0.58^†^
Recreational drug user	14 (20.3%)	4 (30.8%)	3 (16.7%)	7 (18.4%)	0.66^†^
Etiology of PAH (n, %)					
IPAH	30 (43.5%)	4 (30.8%)	8 (44.4%)	18 (47.4%)	0.58
CTD-PAH	24 (34.8%)	8 (61.5%)	5 (27.8%)	11 (29%)	0.08
MA-PAH	8 (11.6%)	0	3 (16.7%)	5 (13.2%)	0.41^†^
HIV-PAH	5 (7.3%)	1 (7.7%)	1 (5.6%)	3 (7.9%)	0.99^†^
CHD-PAH	3 (4.4%)	0	2 (11.1%)	1 (2.6%)	0.25^†^
NYHA class (n, %)					
I	1 (1.5%)	0	0	1 (2.7%)	-
II	15 (22.4%)	4 (33.3%)	3 (16.7%)	8 (21.6%)	-
III	35 (52.2%)	5 (41.7%)	9 (50%)	21 (56.7%)	-
IV	16 (23.9%)	3 (25%)	6 (33.3%)	7 (18.9%)	-
Pulmonary Function Test					
FVC n (Mean ± SD) in L	24 (2.7 ± 1.1)	4 (2.4 ± 0.7)	6 (2.9 ± 1.3)	14 (2.7 ± 1.2)	0.82^‡^
% predicted FVC ± SD	71.7 ± 19.2	67.9 ± 21.8	71.4 ± 16.2	73 ± 20.9	0.9^‡^
FEV1 n (Mean ± SD) in L	24 (2.1 ± 0.8)	4 (2 ± 0.6)	6 (2.2 ± 1.1)	14 (2 ± 0.9)	0.9^‡^
% predicted FEV1 ± SD	71 ± 16.6	69.4 ± 15.7	73.1 ± 14.3	70.5 ± 18.7	0.94^‡^
DLCO mL/min/mmHg n (Mean ± SD)	18 (11.3 ± 7.5)	2 (10.4 ± 1.4)	5 (9 ± 8.9)	11 (12.6 ± 7.7)	0.68^‡^
% predicted DLCO ± SD	59.2 ± 24.6	39.1 ± 3.6	56.6 ± 22.8	64 ± 26.6	0.43^‡^
PAH treatment (n, %)					
IV	12 (17.4%)	1 (7.7%)	4 (22.2%)	7 (18.4%)	0.57^†^
SQ	2 (2.9%)	1 (7.7%)	0	1 (2.6%)	0.41^†^
Diuretics	48 (69.6%)	10 (76.9%)	11 (61.1%)	27 (71.1%)	0.61

Functional capacity and 6MWT

At the time of diagnosis, 51 (76.12%) patients were identified as having advanced functional classes (NYHA classes 3 and 4). There were no statistically significant differences in functional class observed among the three groups, both at the time of diagnosis and three months post-initiation of PAH therapy (Table 1). Additionally, there were no statistically significant variations in the 6MWT among the three BMI groups at the time of diagnosis. Following the onset of PAH treatment over a three-month period, the 6MWT demonstrated an overall improvement, with the most substantial enhancement observed in the obese group at approximately 43 meters, compared to 22 meters in the overweight group and 25 meters in the normal BMI group (Table 2).

**Table 2:** The 6-minute walk test, NT-proBNP, and REVEAL lite at diagnosis and three to six months following the initiation of PAH treatment ^‡^One-way ANOVA ^¥^Kruskal-Wallis H test Significant if p<0.05 6MWT: 6-minute walk test; BMI: body mass index

Parameters	Total (n=69)		BMI <25 (n=13)		BMI 25–30 (n=18)		BMI >30 (n=38)		p-value
	n	Mean ± SD	n	Mean ± SD	n	Mean ± SD	n	Mean ± SD	
6MWT at diagnosis (m)	61	276.1 ± 132	12	298.1 ± 133.6	16	320 ± 152.3	33	246.7 ± 116.7	0.16^‡^
6MWT at 3-6 mo after treatment (m)	58	308.6 ± 149.7	11	323 ± 166.5	14	342.3 ± 157.6	33	289.5 ± 142.2	0.52^‡^
NT-proBNP at diagnosis (pg/mL)	62	1386.5 (599–2728)	11	1898.1 (1127–4871)	16	744 (332.5–1269)	35	1590 (757–3159)	0.04^¥^
NT-proBNP at 3-6 mo after treatment (pg/mL)	56	601.5 (328.7–1810.7)	10	869.5 (398–3730)	14	381.6 (188–525)	32	793.9 (385–1954.7)	0.05^¥^
REVEAL lite at diagnosis	61	9 (7–10)	12	8.5 (6–10.5)	16	7 (6–10)	33	9 (7–11)	0.49^¥^
REVEAL lite at 3-6 mo after treatment	55	8 (4–9)	11	8 (5–8)	12	7 (2–9)	32	8 (4.5–9)	0.86^¥^

Pulmonary function test

Pulmonary function tests at diagnosis showed no significant differences in forced vital capacity (FVC), forced expiratory volume in one second (FEV₁), or diffusing capacity for carbon monoxide (DLCO) among the normal, overweight, and obese groups (Table 1), indicating similar baseline lung function across BMI categories.

NT-proBNP

Baseline NT-proBNP levels at the time of diagnosis showed statistical significance among the three groups (p=0.03). The normal BMI group recorded the highest levels at 1898 pg/mL, contrasting with the lowest levels observed in the overweight group at 744 pg/mL (Table 2). Additionally, the obese group displayed an NT-proBNP level of 1590 pg/mL. After the initiation of PAH treatment at three months, there was a general downward trend in average NT-proBNP levels across all groups (Table 2).

REVEAL lite score

At the time of diagnosis, REVEAL lite was highest in the obese group and lowest in the overweight group. After treatment, REVEAL lite decreased for both the obese and normal BMI groups. However, it remained stable for the overweight group, which maintained the lowest score at 7 (Table 2).

Parenteral therapy

In the management of PAH, 14 (20%) patients were receiving parenteral prostaglandin analog therapy (Table 1). Importantly, there were no statistically significant differences in the use of this treatment across the three BMI groups, indicating comparable administration of PAH therapies among them.

Hemodynamics from echocardiography and RHC

According to echocardiography findings, the obese group exhibited a statistically significant higher proportion of patients with severe RV dysfunction at 30.56%, surpassing the rates of 12.5% in the overweight group and 8.33% in the normal BMI group. Furthermore, pulmonary artery systolic pressure (PASP) measured from echocardiography was highest in the obese group at 74.85 mmHg, compared to 67.27 mmHg in the normal BMI group and 69.85 mmHg in the overweight group. It is noteworthy, however, that despite these differences, they did not achieve statistical significance.

Hemodynamic findings from RHC at diagnosis revealed that the obese group displayed unfavorable measures, with statistically significant elevations in systolic pulmonary artery pressure (SPAP), mean pulmonary artery pressure (mPAP), right atrial pressure (RAP), PCWP, and the lowest pulmonary artery pulsatility index (PAPi), in comparison to the other two groups. Additionally, the cardiac index (CI) was observed to be lower in the obese group than in the other two groups, although this difference did not reach statistical significance (Table 3).

**Table 3:** Hemodynamic findings from echocardiography and right heart catheterization *By the thermodilution technique ^‡^One-way ANOVA ^¥^Kruskal-Wallis H test Chi-square test. Significant if p<0.05 RV: right ventricular; PASP: pulmonary artery systolic pressure; RHC: right heart catheterization; MPAP: mean pulmonary artery pressure; RAP: right atrial pressure; PCWP: pulmonary capillary wedge pressure; PVR: pulmonary vascular resistance; PAPI: pulmonary artery pulsatility index

Method	Total (n=69)		BMI <25 (n=13)		BMI 25-30 (n=18)			BMI >30 (n=38)		p-value
Echo										
RV Dysfunction	64									0.01^¥^
None, n (%)	-	26 (40.62%)	-	9 (75%)	-	8 (50%)	-		9 (25%)	-
Mild	-	9 (14.06%)	-	1 (8.33%)	-	1 (6.25%)	-		7 (19.44%)	-
Moderate	-	15 (23.44%)	-	1 (8.33%)	-	5 (31.25%)	-		9 (25%)	-
Severe	-	14 (21.88%)	-	1 (8.33%)	-	2 (12.50%)	-		11 (30.56%)	-
PASP n (Mean ± SD)	59	72.33 ± 21.96	11	67.27 ± 21.14	13	69.85 ± 13.33	35		74.85 ± 24.72	0.55‡
Pericardial effusion n (Mean ± SD)	22	34.38	7	58.33	3	18.75	12		33.33	0.09
RHC, n (Mean ± SD)										
MPAP	61	50.04 ± 12.87	13	43 ± 8.85	14	48.16 ± 8.12	34		53.5 ± 14.6	0.03^‡^
RAP	50	10 (5–18)	11	4 (2–8)	10	10 (7–12)	29		14.3 (8–20)	0.01^¥^
PCWP	60	12 (8.5–15)	13	10 (8–11)	14	10 (8–14)	33		14 (11–18)	0.02^¥^
PVR	59	692 (480–1032)	13	678 (530–960)	13	703 (455–1074)	33		692 (517–999)	0.92^¥^
CO*	59	4.28 ± 1.39	13	4.03 ± 1.48	13	4.47 ± 1.31	33		4.31 ± 1.42	0.71^‡^
CI*	54	2.17 ± 0.68	13	2.38 ± 0.88	11	2.42 ± 0.52	30		1.99 ± 0.6	0.09^‡^
PAPI	48	5.45 (3.05–9.79)	11	10 (7.25–13)	10	5.45 (3.25–5.86)	27		3.57 (2.5–8)	0.03^¥^

Survival

**Figure 1:**
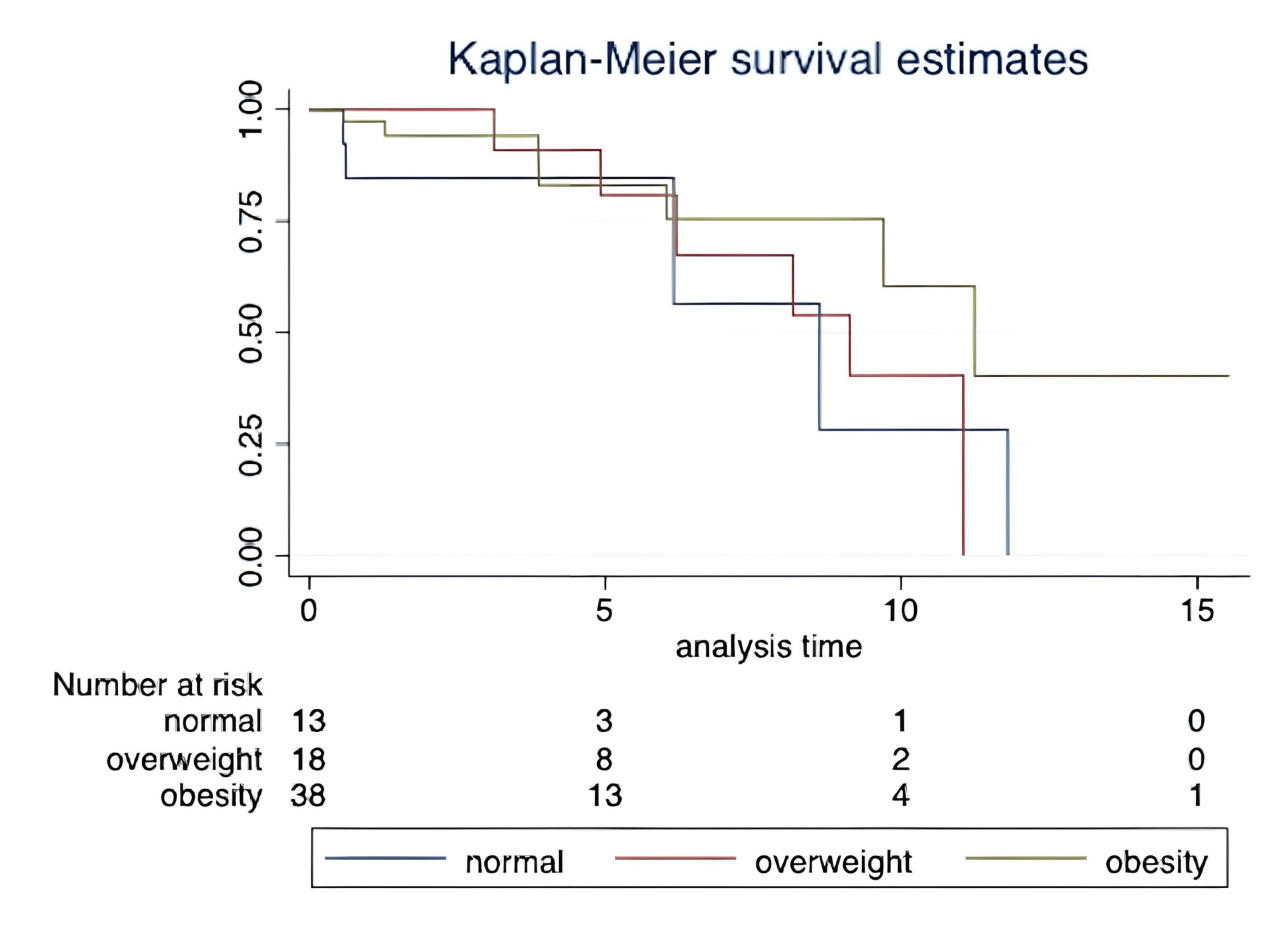
Kaplan-Meier survival curves for three BMI categories (normal, overweight, and obese) in PAH patients. The curves demonstrate survival trends across the groups, with the obese group showing a trend toward improved survival compared with the normal and overweight groups, though the differences were not statistically significant. Normal BMI: Median survival time of 8.3 years; survival rate of 28.2% (95% CI: 0.0107–0.7028) by the end of the follow-up period. Used as the reference group. Overweight: Median survival time of 9.5 years; survival rate of 40.4% (95% CI: 0.0979–0.7023). Hazard ratio (HR) of 0.73 (95% CI: 0.21–2.52), p = 0.619, compared to normal BMI. Obese: Median survival time of 12.2 years; survival rate of 41.4% (95% CI: 0.0751–0.7399). HR of 0.41 (95% CI: 0.13–1.30), p = 0.131, compared to normal BMI.

Kaplan-Meier survival analysis revealed median survival times of approximately 8.3 years in the normal BMI group, 9.5 years in the overweight group, and 12.2 years in the obese group. The maximum follow-up duration for the cohort extended up to 15 years, with varying levels of censoring across groups. At the end of the study period, the survival rates were 28.2% (95% CI: 0.0107-0.7028) for the normal BMI group, 40.4% (95% CI: 0.0979-0.7023) for the overweight group, and 41.4% (95% CI: 0.0751-0.7399) for the obese group, suggesting a trend toward improved survival in the obese group (Figure 1).

However, comparisons of survival curves using the log-rank test showed no statistically significant differences between groups. Hazard ratios further indicated non-significant trends, with an HR of 0.73 (95% CI: 0.21-2.52, p = 0.619) for overweight versus normal BMI and an HR of 0.41 (95% CI: 0.13-1.30, p = 0.131) for obesity versus normal BMI. These results suggest a potential survival advantage for obese patients with PAH, though this finding was not statistically significant.

## Discussion

This is a single-center, retrospective, observational study that examined baseline characteristics, functional capacity, hemodynamic findings, and survival estimates across three BMI categories in our PAH population. Our analysis provides additional insights into the impact of obesity on PAH within our distinct patient population. Our study has a higher proportion of participants classified as obese, with 55.1% meeting this criterion by BMI, compared to 32% in the REVEAL study and 35.7% in the SVPU cohort [5,9]. Similar to those trials, our population was predominantly female (72%) across all BMI groups. The most common etiology for PAH varied, with CTD-PAH prevalent in normal-weight patients, while obese patients predominantly suffered from IPAH. There were no statistically significant differences in comorbidities among BMI groups, such as CKD, DM, CAD, or OSA, but there was a higher incidence of OSA in our obese population. Similarly, there was no statistically significant difference in social history obtained from all subjects, such as smoking, alcohol use, or recreational drug use. There were no statistically significant differences in functional class and 6MWT at diagnosis among the three BMI groups.

In terms of echocardiogram findings, our cohort of obese patients displayed notable features, including the highest prevalence of RV dysfunction, pericardial effusions, and elevated PASP. Hemodynamically, the obese population exhibited elevated RAP, mPAP, and PCWP, along with the lowest PAPi. Despite these concerning echocardiogram and RHC findings, the obese group demonstrated the most favorable trend in survival outcome, although this did not reach statistical significance.

Our findings are consistent with the SVPU trial [9] and the French Pulmonary Hypertension Network registry [13], which also found no statistically significant difference in survival between obese and non-obese patients. However, our results diverge from the US-based REVEAL study, which reported an overall reduced risk of death among obese patients [5].

The obesity paradox remains an enigma, with various theories attempting to elucidate why obese patients may experience better survival outcomes in the context of PAH. One such theory posits that obese patients might benefit from lead-time bias, wherein they are diagnosed and treated at an earlier stage of their disease compared to individuals in other BMI groups [13]. Additionally, obese patients often present with a higher number of comorbid conditions and are more likely to have an established relationship with a healthcare provider, potentially facilitating the early detection and prevention of disease progression.

Previous studies have proposed several mechanisms linking obesity to PAH, including inflammation, oxidative stress, endothelial dysfunction, increased leptin levels, reduced adiponectin levels, and altered estrogen metabolism [1]. It is hypothesized that the improved survival observed in obese patients may be due to PAH-specific treatments that have anti-inflammatory, antiproliferative, and oxidative-stress-reducing effects [2]. As a result, obese patients might benefit more from these therapies, potentially explaining the obesity paradox in PAH [1,12,16].

The uneven distribution of females across BMI groups could contribute to the more favorable survival observed in the obese group. Our studies found a higher percentage of females in the obesity group. Previous research indicates that female PAH patients tend to experience better outcomes despite being more predisposed to developing PAH compared to males, a phenomenon known as the estrogen paradox [1,17]. Consequently, the higher proportion of females in the obese group could potentially explain the better survival observed in this group.

Variations in the causes of PAH and the distribution of PAH subtypes across patient groups may help explain the differences in survival among BMI categories. Our findings showed that 60% of obese patients had idiopathic PAH (IPAH), while 62% of patients in the BMI <25 group had CTD-PAH. As previous research has consistently shown a poorer prognosis for CTD-PAH compared to IPAH [3,18], the overall survival advantage observed in our population may be more closely linked to the specific PAH subtype rather than to obesity alone.

Beyond PAH subtypes, certain comorbid conditions prevalent in the obese subgroup may also play a role in survival outcomes. Notably, 31.6% of obese patients in our population had OSA. A previous study proposed that apnea during sleep could trigger cardioprotective effects resembling ischemic preconditioning [19]. This phenomenon leverages the heart's ability to 'condition' itself to withstand an ischemia-reperfusion injury, potentially contributing to longer survival. This cardioprotective mechanism associated with OSA may partly explain the favorable survival trend observed in obese PAH patients [19,20].

While factors such as PAH subtypes and comorbid conditions, such as OSA, may contribute to survival differences, several other factors may explain the lack of statistically significant survival differences across BMI groups in our study. Firstly, our relatively small sample size may limit the power to detect meaningful differences. Additionally, our cohort includes a high proportion of African American individuals (44.8%), compared to only 13% in the REVEAL study and 17.2% in a meta-analysis by Agarwal et al. [21]. This reflects Louisiana's high obesity rate [14] and the unique demographics of the area where our study was conducted. Previous research has highlighted ethnic disparities in PAH outcomes, likely influenced by genetic, pharmacogenetic, and cardiovascular variations, such as reduced RV mass, higher prevalence of RV dysfunction, and socioeconomic challenges [22-24]. These complex, multifactorial influences may help explain the non-significant survival differences observed in our BMI-diverse population.

Our study, while offering valuable insights, has limitations that should be considered. As a single-center study with a relatively small sample size, the generalizability of our findings to broader populations may be limited. The modest sample size also reduces the statistical power needed to detect significant differences across BMI groups and restricts our ability to adjust for potential confounding factors. Additionally, the retrospective, observational design lacks the longitudinal perspective necessary to examine the effects of changes in BMI over time, which could provide important information on the impact of weight fluctuations on PAH outcomes. These limitations highlight the need for future multicenter, prospective studies with larger cohorts and longer follow-ups to validate our findings and explore the dynamic effects of BMI on PAH progression and survival.

The rising prevalence of obesity among PAH patients underscores the need for a thorough investigation into how weight impacts disease management and outcomes. Current clinical trials often exclude obese patients, contributing to a data gap and inconsistent findings regarding PAH outcomes in this population. Future research should explore the effects of body weight on PAH drug pharmacokinetics to guide weight-specific treatment strategies. Additionally, advancing our understanding of prognosis in obese PAH patients is crucial, particularly in evaluating the accuracy of biomarkers like BNP, which may not apply universally due to variations in obese populations. Adopting a more inclusive approach in future studies, with a focus on diverse BMI categories, will help clarify the relationship between body weight and survival in PAH patients. Such research is essential to developing personalized, effective management strategies for PAH across varied weight profiles.

## Conclusions

In conclusion, our study contributes to the expanding literature on the obesity paradox in PAH, showing that obese PAH patients may exhibit a trend toward favorable survival outcomes despite more adverse hemodynamic profiles. This paradox underscores the importance of considering BMI and related comorbidities when evaluating PAH prognosis and management strategies. The unique demographic and clinical characteristics of our cohort, including a high prevalence of obesity, provide a distinct perspective on the potential protective mechanisms in obese patients, such as the role of early diagnosis and response to PAH-specific therapies. These findings highlight the need for future multi-center studies with larger, more diverse populations to further elucidate the complex relationship between obesity and PAH outcomes, ultimately guiding more tailored and effective care for this population.
